# Clinically relevant nanodosimetric simulation of DNA damage complexity from photons and protons†

**DOI:** 10.1039/c8ra10168j

**Published:** 2019-02-28

**Authors:** N. T. Henthorn, J. W. Warmenhoven, M. Sotiropoulos, A. H. Aitkenhead, E. A. K. Smith, S. P. Ingram, N. F. Kirkby, A. L. Chadwick, N. G. Burnet, R. I. Mackay, K. J. Kirkby, M. J. Merchant

**Affiliations:** Division of Cancer Sciences, School of Medical Sciences, Faculty of Biology, Medicine and Health, The University of Manchester UK nicholas.henthorn@manchester.ac.uk; The Christie NHS Foundation Trust, Manchester Academic Health Science Centre Manchester UK; Christie Medical Physics and Engineering, The Christie NHS Foundation Trust Manchester UK

## Abstract

Relative Biological Effectiveness (RBE), the ratio of doses between radiation modalities to produce the same biological endpoint, is a controversial and important topic in proton therapy. A number of phenomenological models incorporate variable RBE as a function of Linear Energy Transfer (LET), though a lack of mechanistic description limits their applicability. In this work we take a different approach, using a track structure model employing fundamental physics and chemistry to make predictions of proton and photon induced DNA damage, the first step in the mechanism of radiation-induced cell death. We apply this model to a proton therapy clinical case showing, for the first time, predictions of DNA damage on a patient treatment plan. Our model predictions are for an idealised cell and are applied to an ependymoma case, at this stage without any cell specific parameters. By comparing to similar predictions for photons, we present a voxel-wise RBE of DNA damage complexity. This RBE of damage complexity shows similar trends to the expected RBE for cell kill, implying that damage complexity is an important factor in DNA repair and therefore biological effect.

## Abstract 2

Relative Biological Effectiveness (RBE) is a controversial and important topic in proton therapy. This work uses Monte Carlo simulations of DNA damage for protons and photons to probe this phenomenon, providing a plausible mechanistic understanding.

## Graphical abstract

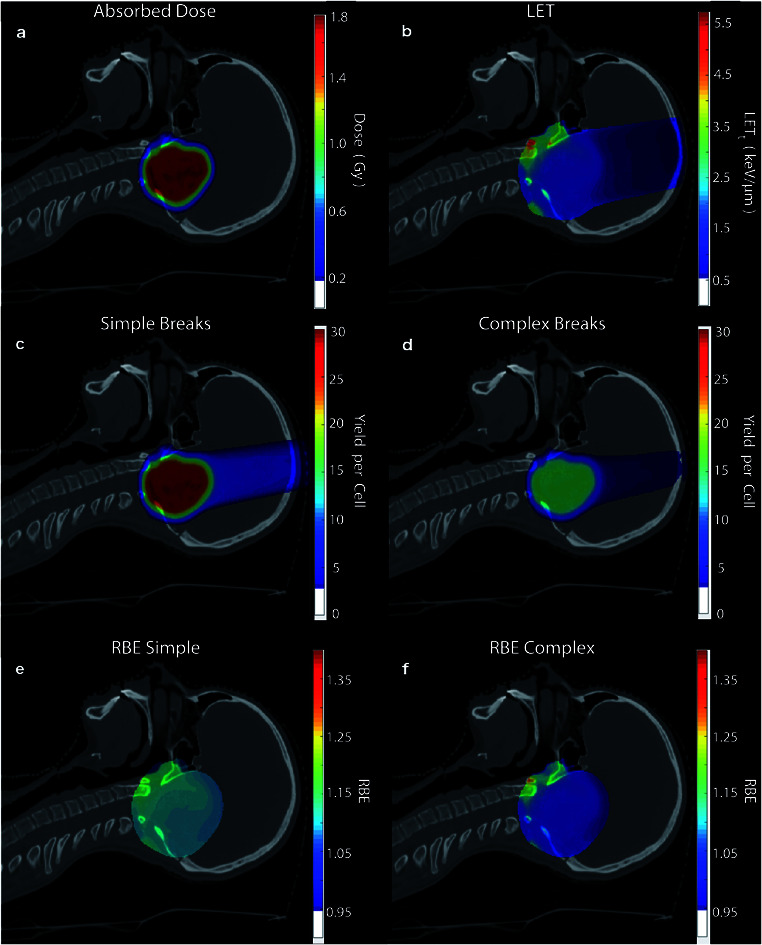

Proton therapy offers many potential benefits for cancer treatment due to the favourable dose depth profile, characterised by the Bragg peak. This modality appears to offer a superior normal tissue sparing effect compared to photons. However, most conventional radiotherapy has been delivered with high energy photons and a wealth of knowledge exists with this modality, with decades of data informing the optimal dose prescription and normal tissue dose constraints.^1^ To utilise photon experience in proton therapy a dose conversion is applied through use of the Relative Biological Effectiveness (RBE). RBE is defined as the ratio of doses between a reference radiation and a test radiation to achieve the same biological effect. One common definition of the biological effect is 10% cell survival measured through clonogenic assays. For protons a constant value of RBE = 1.1 is in clinical use,^2^ stating that for the same dose protons are 10% more effective at cell killing than photons. However, there is considerable variance in the experimental data informing RBE,^3^ with the value chosen as a conservative compromise. It has been shown *in vitro* that proton RBE is not constant but instead depends on many factors, including Linear Energy Transfer (LET), dose, and tissue type.^3^ More recently there has been emerging clinical evidence that a static RBE may lead to normal tissue toxicities for a small number of patients, characterised through image changes^4,5^ and brainstem necrosis.^6^ To encompass variable RBE a number of phenomenological models have been proposed.^7–9^ These models link the photon radiosensitivity parameters of the linear-quadratic cell survival model to parameters of the proton beam, such as dose and LET. The models can reproduce the heightened cell kill at increasing LET. However, it is not possible for this type of model to directly suggest a mechanism for the effect. The lack of mechanistic understanding limits the applicability of these models when they are used beyond their fitted range. Furthermore, it has been argued that LET alone is not an adequate parameter for describing RBE, particularly when considering different ion species.^10^

Alternative to the phenomenological approaches are mechanistic computational models. A branch of these mechanistic models rely on the Monte Carlo method to simulate radiation effects in the cell. A number of simulation frameworks exist to investigate DNA damage and repair at the cellular level.^11–15^ The consensus in the field is that radiation induced cell death is a function of DNA damage and the efficacy of the resultant repair pathways. Isolating the damage and repair aspects *in silico* allows for the determination of dependencies between the two. To investigate mechanisms such as these the model must begin from no prior assumptions, such as an LET dependence. Instead the link between radiation quality and biological outcome should be emergent from the results. The presence of emergent behaviour gives confidence that mechanisms have been correctly described, and that the model can be extrapolated to investigate beyond experimental data.

Often simulations will score the yield of Double Strand Breaks (DSBs), the most toxic damage, as well as aspects of the induced DSBs such as complexity. Clinically relevant conclusions have been drawn with this methodology, for example the importance of proximity effects^16^ and break complexity.^17^ It has been shown *in silico* that DSBs are produced more proximal with increasing LET.^18^ Closer proximity between DSBs promotes misrepair, which can manifest later as chromosome aberrations.^19,20^ This is important when considering the biological effect at the distal edge of the proton Bragg peak, where LET is highest.

Experimental *in vitro* evidence has led to the hypothesis that complex damage is more difficult to repair^21^ and may play a role in determining repair pathway choice.^22^ If DNA damage persists at the end of the cell cycle then the unrepaired damage can cause cell cycle arrest, leading to cell death or senescence.^23^ As such, it is hypothesised that the initial DNA damage pattern is strongly correlated to the early biological outcomes. Efforts are underway to measure and quantify this effect both experimentally and through simulation, broadly grouped under the field of nanodosimetry.^24^ Experimentally, a number of nanodosimeters are in operation.^25–28^ In nanodosimetry clusters of ionisations at the nanoscale are detected in gas, scaled to represent water. The metric of note is the Ionisation Cluster Size Distribution (ICSD), describing the number of ionisations forming a cluster,^29^ which is characteristic for a given radiation quality.^30^ It is hypothesised that the ICSD is related, or equivalent, to the complexity of DSB that would be created by the radiation in a cell.^31,32^ This nanodosimetric scoring has begun to show clinical relevance, with studies now proposing an implementation into treatment planning systems^33^ (E. Smith *et al.*, in preparation). Biologically the case may be more complex since the nanodosimetric method only accounts for direct physical damage and neglects indirect damage, resulting from free radical production. The availability of 4D track structure simulation now offers the opportunity to model the complete DNA damage mechanisms.^11,34,35^ Modelling of indirect damage is particularly important when considering hypoxia, which is common to many tumour types. This decreased oxygen concentration has an impact on the DNA damage, the biological response, and treatment outcomes.^36–38^

The work presented here spans many scales, starting with investigation of DNA damage mechanisms and ending with the application of the model to a proton therapy case. We present the results of track structure simulations, using Geant4-DNA, to score DNA damage at the nanometre scale. By comparing simulation to experimental results of plasmid irradiation in the literature we determine the optimal combination of DNA geometry model and method for scoring direct DNA damage. We include indirect damage in the simulation by tracking the creation, diffusion, and interaction of free radicals through the Geant4-DNA chemistry module.^39^ These mechanisms of direct and indirect damage are used to predict DNA damage complexity by simulating the irradiation of the chromatin fibre and a model of the cell nucleus. Throughout the work correlations are drawn that relate the yield and complexity of DNA damage to track averaged Linear Energy Transfer (LET_t_) and dose. The clinical relevance of these correlations is demonstrated with a simulated treatment of an ependymoma case, planned using a research version of Varian's Eclipse™ treatment planning system. Here, yields for given types of DSB complexity are predicted. To our knowledge this is the first time that a direct prediction of the underlying biological damage at the DNA level has been applied to a proton radiotherapy case. By comparing to similar predictions for photons an RBE of damage type is shown. The predictions of RBE are similar in trend when compared to the RBE of cell kill predicted by the phenomenological models. Although we do not currently model DNA repair in this work the trends imply a role for damage complexity in cell kill. This type of scoring is the first step on the path towards biologically optimised radiotherapy.

## Methods

### Models of DNA geometry

**Fig. 1:**
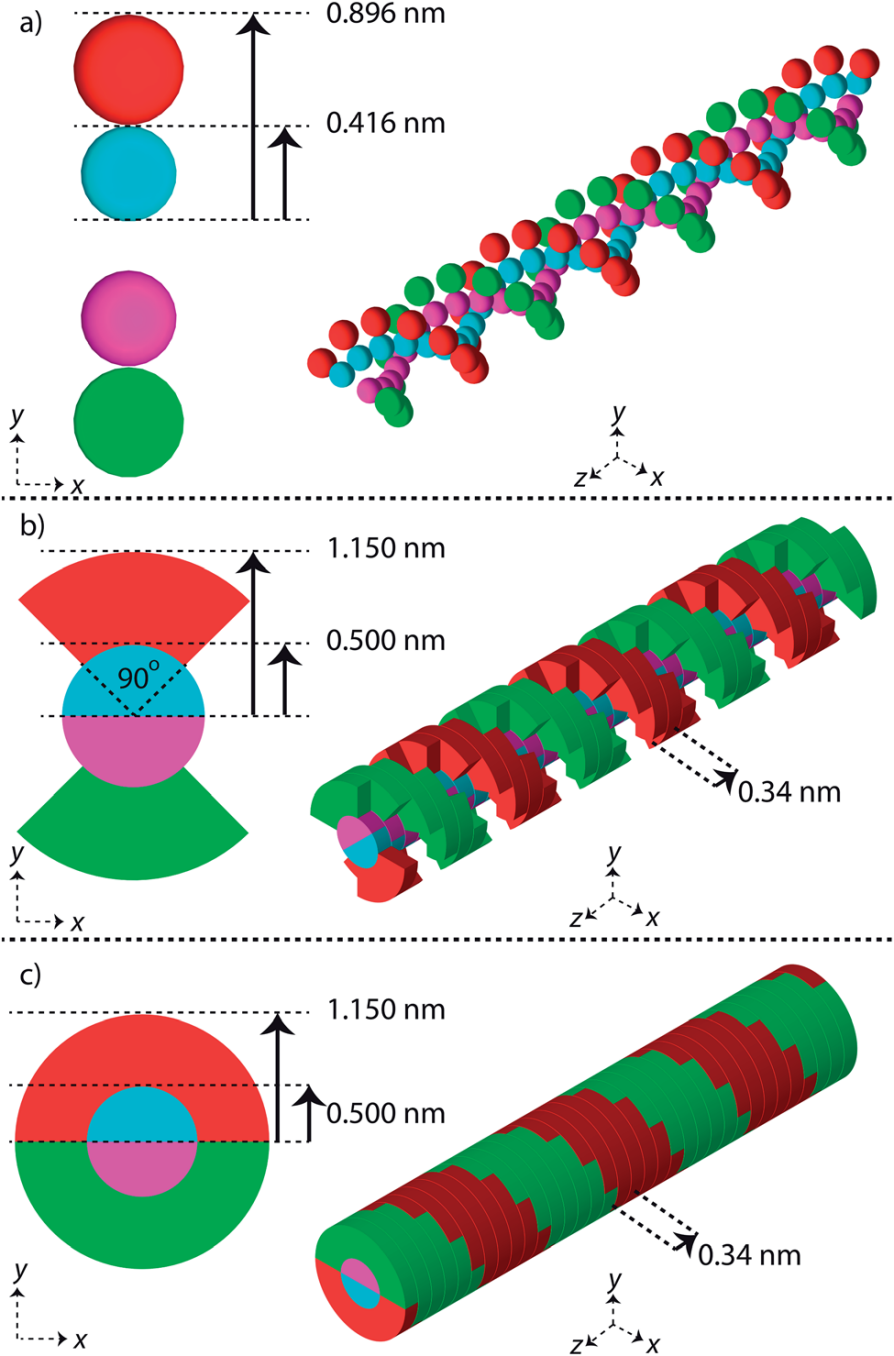
Linear segments of the simple DNA double helix geometries implemented in this work. Each backbone and base is constructed from a discrete volume and assigned an ID number equivalent to the base pair. The first column of images shows a cross section through the double helix, the second column shows a 30 bp segment of the double helix. (a) Sphere, (b) QuartCyl, (c) HalfCyl.

Three simple geometric models of the DNA double helix have been implemented in this work (Fig. 1). These models have previously been used in published simulation work with the aim of predicting DNA damage.^40–42^ For each model the DNA sugar-phosphate backbone and the DNA base volume are formed by single discrete volumes. The DNA volumes are assigned an identification number equivalent to their position along the genome, allowing for exact determination of base pair (bp) separation between damaged volumes.

The first geometrical model of the DNA double helix (Spheres) is based on our previous work,^40^ where the backbones and bases are constructed as spheres (Fig. 1a). In this model, the backbone has a radius of 0.240 nm and the base has a radius of 0.208 nm. The second DNA geometry (QuartCyl) was proposed by Bernal and Liendo^41^ (Fig. 1b). Here, the base and backbone are formed with a half cylinder and quarter cylinder respectively. The base has a radius of 0.5 nm and thickness of 0.34 nm. The backbone has a full radius of 1.15 nm, with a cut away section for the base. The model used here differs slightly from the original publication by Bernal and Liendo. We do not build the double helix with a major and minor groove, instead placing the nucleotides directly opposite each other. The third geometry (HalfCyl) was proposed by Charlton, Nikjoo, and Humm^42^ (Fig. 1c). Each base is formed from a half cylinder, with a radius of 0.5 nm and a thickness of 0.34 nm. The bases from each strand are built opposite each other and are surrounded by half cylinders representing the backbone, with a cut away section for the base. The backbone has a full radius of 1.15 nm and a thickness of 0.34 nm. Most recently this model has been used as part of a validation study for the TOPAS-nBio code.^14^

For each DNA model a rotation of 36 degrees around the central axis is applied between successive base pairs, achieving a full turn of the double helix every 10 bp. All DNA volumes in the simulation are made from liquid water, with a density of 1.407 g cm^−3^.^43^

### Track structure simulation

The Monte Carlo toolkit Geant4 (version 10-02-patch01),^44^ with the Geant4-DNA extension^45,46^ was used to simulate the transport and interaction of mono-energetic protons with the volumes described in this work. Within Geant4-DNA particles are tracked using an event-by-event method, where each track is composed of a series of particle steps. Currently, the Geant4-DNA physics list (G4EmDNAPhysics) is limited to simulation in liquid water targets but does allow for changes in water density. Representing biological materials with water is a standard assumption in radiobiological Monte Carlo studies.^47^

For comparison, photon induced DNA damage is investigated by exposing the simulated biological targets to the secondary electrons produced by a Co-60 source. To determine the secondary electron energy spectrum, Geant4 (G4EmStandardPhysics) is used to simulate the transport of 1.17 MeV and 1.33 MeV photons through a 10 × 10 × 10 mm^3^ water box, with photon intensity equal to the decay scheme of Co-60. The energy of any secondary electrons created by the primary photon is recorded and binned to create an energy probability distribution. The distribution is used to randomly select an electron energy for simulation with the biological targets.

### Direct and indirect DNA damage

A number of methods have been proposed to convert energy depositions into DNA damage. We consider three methods in this work; an energy range, a threshold energy, and ionisation events.

In the first method, it is assumed that the total energy deposited within a DNA volume has a probability of causing damage (energy range). A linear probability is applied, between the energy range of 5 eV and 37.5 eV. This range has been informed by experimental results of DNA strand breaks from low energy electrons and photons.^48^ The energy depositions in a DNA volume are summed for a primary particle and its associated secondaries. The second method uses a threshold energy for damage (energy threshold).^42^ In this method DNA volumes that receive energy depositions of more than 17.5 eV are considered damaged, with the threshold value reported by Nikjoo *et al.*^49^ The third method scores ionisation events within the DNA volumes (ionisation). This approach has a relevance for the emerging field of nanodosimetry, where it is hypothesised that clusters of ionisations at the nanoscale are related to early biological outcomes.^24^ For this method the DNA volumes in which an ionisation event occurs are considered damaged.

Following the simulation of the physical interactions the Geant4 chemistry modules are invoked.^39,50^ The yield, species, and position of the free radicals are determined by Geant4-DNA based on interactions of the physical beam with water molecules. The biologically relevant radiochemistry is described in terms of water radiolysis since water constitutes the majority of the cell mass. All of the free radicals are tracked for 1 ns with Brownian diffusion, including chemical reactions with other free radicals. Hydroxyl radicals (OH) are assigned a probability of causing damage to the DNA backbone or base for a step taken in the DNA volume. The probability of OH radicals damaging a base is set to 0.8.^51^ The probability of OH induced DNA backbone damage is fitted to match the estimated ratio of 35 : 65 between direct and indirect damage, for the case of Co-60 irradiation. The estimated ratio between direct and indirect DNA damage was first suggested by Ward.^52^ The assumed ratio has become the standard for the PARTRAC code.^34,53^ If the OH radical meets the probability conditions, then the damage is recorded and the OH track is terminated in the simulation, equivalent to chemical reaction with the DNA leading to a strand break. If the probability condition is not met the OH track is terminated without recording any damage, equivalent to a chemical reaction with no DNA damage. Within this method is the assumption that OH radicals entering a DNA volume always react.

### Damage classification

Following the simulation of the primary and secondary particles, and any associated free radicals, the damaged DNA volumes are analysed by a clustering algorithm. For determination of DSBs the clustering algorithm searches for damaged DNA backbones that are on opposite strands and separated by 10 bp or less.^53^ This can lead to the formation of a cluster containing multiple damages. We assume that this type of damage leads to one DSB, though in reality the situation may be more complex with the potential for multiple breaks and small deletions. It has been suggested that base damage within 1–3 bp of a DSB can interfere with repair,^17,54,55^ to consider this we include damaged bases into a DSB if they are within 3 bp of the extremities of the damage site.^17,54,55^ Any damaged base that is directly attached to a damaged backbone is neglected from the clustering, since it is assumed that this damage will be removed along with the backbone during repair.

The damage classifications are focused on DSB type, since it is hypothesised that these are the lesion type most closely related to cell death.^56^ The classifications are descriptive of the early physico-chemical damage rather than later damages arising from the biological response. For example, repair of isolated base damage through the Base Excision Repair (BER) pathway can lead to the creation of a short-lived nucleotide gap.^57^ If this process occurs on the opposite strand to a damaged backbone then a DSB may be induced. The process is considered in this work by identifying isolated backbone and base damages that are on opposite strands separated by 10 bp or less, referred to as potential DSBs. Within this work none of the potential DSBs are converted into DSBs. A full classification of this type of time-dependent biologically induced DSB would be better accounted for in a model of the biological response.

**Fig. 2:**
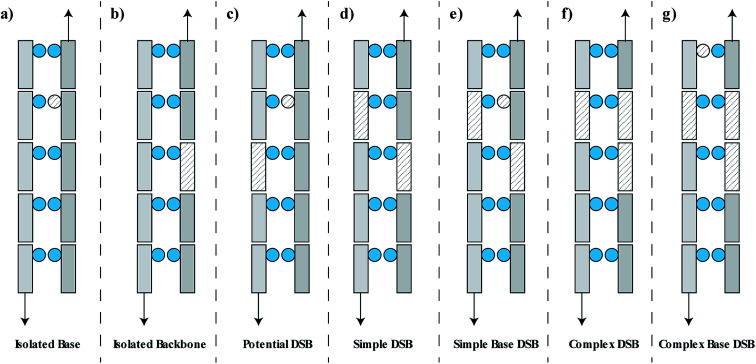
Schematic representation of the DNA damage classifications used in this work. DNA backbones are shown as rectangles, DNA bases are shown as circles. Damaged volumes are filled with dashed lines. (a) Isolated base damage, (b) isolated backbone damage (SSB), (c) potential DSB, (d) simple DSB with damaged backbones on opposite strands separated by less than 10 bp, (e) simple DSB with at least one associated base damage within 3 bp of the DSB ends, (f) complex DSB with multiple associated damaged backbones, (g) complex DSB with at least one associated base damage.

The damage is classified as one of seven groups, shown schematically in Fig. 2. These classifications include an isolated base damage (Fig. 2a), an isolated backbone damage (Fig. 2b), a potential DSB (Fig. 2c), a simple DSB with no associated base damage (Fig. 2d), a simple DSB with at least one associated base damage (Fig. 2e), a complex DSB with multiple associated backbones but no associated base damage (Fig. 2f), and a complex DSB with multiple associated backbones and at least one associated base damage (Fig. 2g). We consider the damages shown in Fig. 2e–g as complex DSBs, whilst the damage shown in Fig. 2d is considered to be a simple DSB. Within the literature there is ambiguity in the term “complex damage”. In this work, complexity refers to the degree of clustered damages within the DSB.

### Simulation of plasmid irradiation

A model of the pBR322 plasmid^58^ is implemented in Geant4-DNA to investigate the induction of direct DNA damage, without repair. Here, 4361 bp of DNA are organised to form a closed circular loop, with the DNA constructed from the three different volumes discussed previously. The plasmid is placed on top of a slab of water, representative of a glass coverslip. Water was chosen for the coverslip material to maintain the Geant4-DNA physics tracking throughout the simulation. The coverslip is simulated to account for any backscattered particles. The plasmid DNA is surrounded by a water torus with a diameter of 2.4 nm. All volumes within the simulation, aside from the coverslip, torus, and DNA, are constructed with air.

The proton beam is simulated perpendicular to the plasmid, passing through the plasmid and then the coverslip. Initially the protons are uniformly placed on a disc 1 μm from the plasmid. To match experimental conditions, a dose of 2000 Gy is delivered for each proton energy investigated. The dose is achieved by fixing the number of primary particles and varying the radius of the irradiation disc, *i.e.* the fluence, according to eqn (1).1
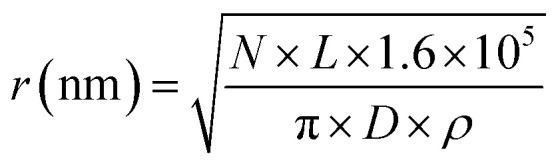
where *N* is the number of primaries (5000), *L* is the proton LET_t_ in units of keV μm^−1^, *D* is the required dose (2000 Gy), and *ρ* is the DNA density (1.407 g cm^−3^).

The induction of DNA damage is determined according to the methods described previously, with only direct damage considered. Yields of SSBs and DSBs are calculated by clustering damaged DNA backbones and reported per Mbp per Gy. The yields are compared to experimental results reported in the literature for dry plasmid irradiation.

### Simulation of chromatin fibre irradiation

A model of the chromatin fibre is implemented; full details of the model have been reported in our previous work.^40^ The chromatin fibre is composed of 102 histones, each wrapped by 1.65 turns of the DNA double helix, arranged in the solenoid conformation.^59^ The organisation of the *in vivo* chromatin fibre is unknown within the literature,^60^ though our previous work compared three proposed geometries to show that neither the yield nor complexity of damage is significantly affected.^40^ The fibre is 198 nm long with a diameter of 37 nm and has a density of 5.7 nucleosomes per 11 nm. A water cylinder is constructed around the fibre, with a length of 203 nm and diameter of 42 nm. In total 18.3 kbp of DNA is built in the chromatin fibre.

Within a cell nucleus the orientation of the fibre is random relative to the proton beam. To account for this in the simulation the mono-energetic proton is initially placed on the surface of the fibre with the direction randomised. Following the simulation of the primary proton, and all associated secondaries and free radicals, the damaged DNA volumes are assessed by a clustering algorithm. This determines the DNA damage per primary, with the assumption that a segment of the chromatin fibre will not be traversed by more than one primary track. Indirect DNA damage is included by tracking OH interactions with the DNA volumes. An OH radical that enters a histone volume is removed from the simulation, recreating the histone free radical scavenging effect.^53^

### Total damage yields in a cell model

The specific pattern of damage predicted by the fibre model is used to populate positional information of damage predicted by simulation of cellular irradiation. The combined model gives the yields and position of each break type for a given dose of protons. Details of the method have been reported in our previous work.^18^ A spherical nucleus (radius of 2.5 μm) is centred in a section of cytoplasm (box with half-length of 5 μm) and a uniform dose is delivered to the nucleus. 15% of energy depositions in the nucleus are recorded, with the percentage chosen to reproduce DSB yields from the literature.^61^ The accepted energy depositions are converted to strand breaks based on the amount of energy deposited, with the probability scaling linearly between 5.0 and 37.5 eV. The strand breaks are then randomly assigned to strand one or two of the double helix. A modified DBSCAN algorithm^62^ searches for clusters amongst the strand breaks, given the condition that strand breaks must be on opposite strands and separated by 3.4 nm or less, equivalent to the separation of 10 bp. The conversion of energy depositions into DSBs follows a similar methodology proposed by Francis *et al.*^63^ The details of damages associated to the DSB, the nucleotide resolution of the break structure, is then populated by randomly sampling data from the chromatin fibre simulations at matching primary particle energy. Isolated strand breaks determined by the cell model are populated with either isolated backbones, isolated bases, or potential DSBs from the fibre model.

The cell and chromatin fibre models can also predict photon induced DNA damage. A large number of photons are required to deliver a given dose. Due to the large number of photons it is assumed that a homogeneous dose is delivered across the nucleus, and as such DSBs can be randomly placed, this assumption dramatically reduces simulation time. The DSB yield is assumed to follow a Poisson distribution with an average of 4.2 DSBs/Gbp/Gy/cell, where the average is determined to fit the DSB yield and LET relation of protons (assuming Co-60 LET_t_ = 0.2 keV μm^−1^). The SSB yield is predicted from the estimated SSB to DSB ratio, between 25–40 for sparsely ionising radiation.^64^ This damage is populated with the specific complexity data from the Co-60 irradiated chromatin fibre.

## Results

### Direct DNA damage – plasmid irradiation

**Fig. 3:**
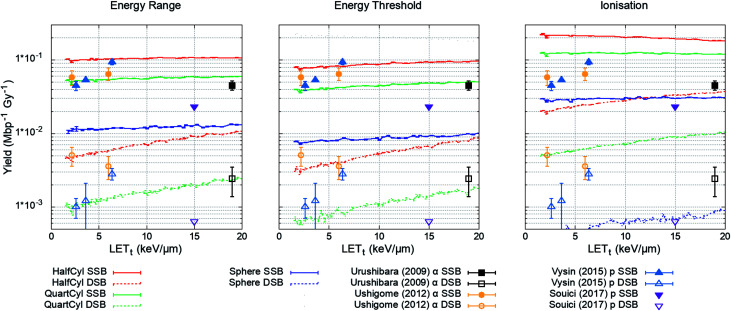
Yield of DSBs (dashed lines) and SSBs (solid line) predicted by the simulation as a function of proton LET_t_. Simulation error bars show the standard error in the mean between 10^4^ repeats of 2000 Gy per LET_t_. Experimental data is replotted from ref. 65–68, here DSBs are shown as open symbols and SSBs are shown as closed symbols. All combinations of geometry and damage model reproduce the magnitude of experimental data, except the ‘Sphere’ model, which significantly underestimates damage yields.

The plasmid model was constructed in Geant4 with DNA volumes selected as either spheres, quarter cylinders, or half cylinders (Fig. 1). The induction of DNA damage was scored according to the three methods discussed; an energy range, energy threshold, or ionisations. Only direct DNA damage was considered for the plasmid simulations, with results compared to experimental data of dry plasmids or plasmids with free radical scavengers in the literature. Simulation and literature results are reported per Mbp per unit dose (Fig. 3); a conversion of 650 Da per bp was assumed for studies reporting data per unit mass.

The simulation was designed to match the experimental conditions of Vyšín *et al.*,^65^ where dry pBR322 plasmids were irradiated with 10, 20, or 30 MeV protons. To assess the simulation's predictive accuracy of DNA damage over a greater range of LET_t_, three further studies were included. Similarly, Souici *et al.*^66^ irradiated dry pBR322 with proton LET_t_ relevant to the Bragg peak region. Urushibara *et al.*^67^ and Ushigome *et al.*^68^ studied the direct DNA damage yields by irradiating hydrated pUC18 plasmids with alpha particles.

The investigated combined geometry and damage models reproduce the relative magnitude of SSB and DSB yields seen experimentally, aside from the spherical DNA model (Spheres), which significantly underestimates yields across the LET_t_ range. For the model combinations tested, the quarter cylinder DNA model with backbone damage determined by an energy range most closely reproduces the experimental data.

### Indirect DNA damage – chromatin irradiation

**Fig. 4:**
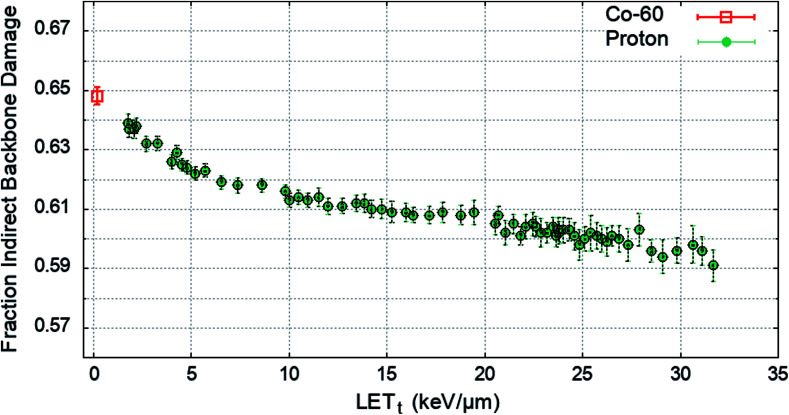
The average fraction of backbone damage due to indirect effects, showing that across the LET_t_ range the majority of damage is from indirect effects. Data is shown for Co-60 (0.2 keV μm^−1^, open symbol) and a range of proton LET_t_ (closed symbols). Error bars show the standard error in the mean between 1.5 × 10^6^ independent primaries.

To determine the impact of indirect effects on DNA damage the chromatin fibre model was irradiated across a range of proton LET_t_, or with an electron energy spectrum from a Co-60 source. The Geant4-DNA chemistry modules were implemented to track the production and motion of free radicals within the geometry. OH radicals crossing a DNA backbone are assigned a probability of inducing damage, *P*_Ind._. The number of backbones damaged by direct and indirect effects are independently summed and the total fraction of strand breaks induced by indirect effects are calculated. *P*_Ind._ was selected to produce 65% total backbone damage from indirect effects^52^ for the case of irradiation with a Co-60 source. *P*_Ind._ was determined to be 0.5, when scored with the quarter cylinder geometry and energy range damage model combination. The fraction of indirect backbone damage for Co-60 and a range of proton LET_t_ is shown in Fig. 4.

A value of *P*_Ind._ = 0.5 leads to indirect effects causing an average fraction of 0.648 ± 0.003 of the total strand breaks for Co-60. The simulation predicts that all values of *P*_Ind._ result in a higher fraction of strand breaks due to indirect effects compared to direct effects, investigated down to *P*_Ind._ = 0.05 (not shown here). The probability of OH damage to DNA bases is set to 0.8 (ref. 51) to account for higher reaction rates.^61^

### Damage complexity – cell irradiation

**Fig. 5:**
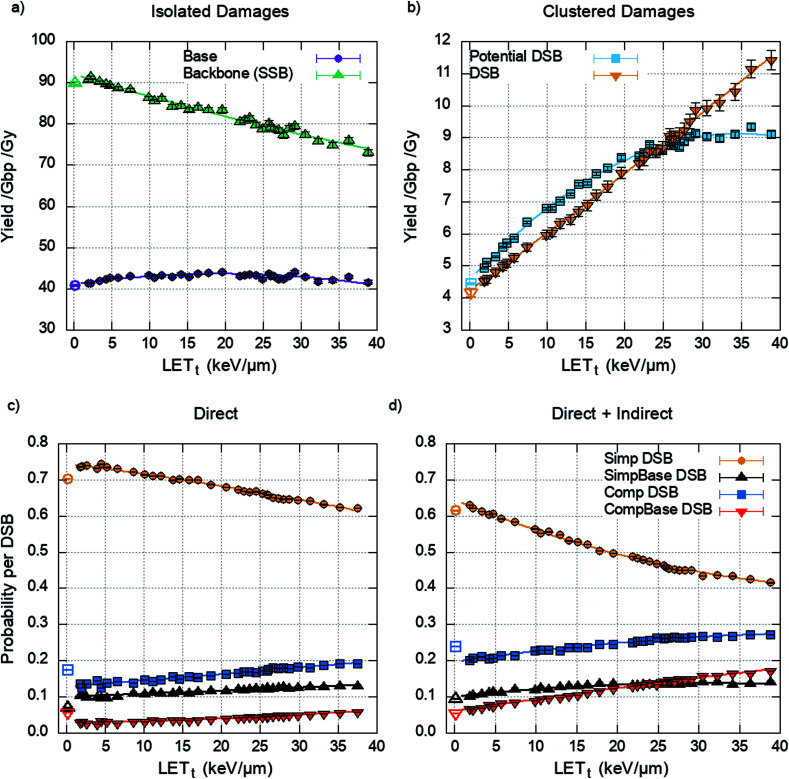
The average yield of DNA damage type per unit dose per Gbp, assuming a genome of 6 Gbp, across the proton LET_t_ range (top panels) and the probability of forming a given type of DSB (bottom panels). (a) Yield of isolated damages. (b) Yield of clustered damages, showing an increase in DSBs and potential DSBs with LET_t_ and a corresponding decrease in the yield of isolated backbones (SSB) as more of these damages are converted into clustered damages. The probability of forming a given type of DSB for (c) direct damage alone, (d) direct and indirect damage. Including indirect effects leads to an increase in more complex breaks, with a corresponding decrease in the simpler form of DSB (Simp DSB). Error bars are the standard error in the mean for 2500 repeats of 1 Gy. Photons are shown as open symbols and protons are shown as closed symbols.

The damage yields determined by the cell model through the clustering of energy depositions are randomly populated with detail from the chromatin fibre simulations, matching proton energy between models. The yields of isolated and clustered damage are shown in Fig. 5a and b respectively.

The DSBs predicted are further categorised into the types of DSBs described previously (Fig. 2). This is presented as a probability per DSB, removing the dependency on the initial DSB yield and therefore dose. The data is generated by the chromatin fibre simulations and as such can determine the DSB types for direct damage alone or direct and indirect damage together. Fig. 5d shows that including indirect effects leads to an increase in complex DSBs, with a corresponding decrease in simple DSBs. This is highlighted between 20–25 keV μm^−1^, where inclusion of indirect effects predicts that the probability of multi-base multi-backbone breaks (CompBase) is greater than multi-base 2 backbone breaks (SimpBase). This increase in complexity, when indirect effects are included, highlights the importance of full damage simulation, since the effect is not seen when only direct damage is simulated (Fig. 5c).

A polynomial fit is applied to the yields shown in Fig. 5b, assuming 6 Gbp of DNA, and the probability of DSB type shown in Fig. 5d, giving the total yield of each DSB category as a function of dose and LET_t_. This correlation, eqn (2), can then be used to predict DSB complexity without the need for simulation.

2 Yield(*D*, *L*) = *D* × (*a* × *L*^2^ + *b* × *L* + *c*)

where *D* is the proton dose in units of Gy and *L* is the track averaged LET in units of keV μm^−1^. The parameters of eqn (2), as well as their asymptotic standard errors, are presented in Table 1.

**Table 1:** The fitted parameters of eqn (2), including the asymptotic standard error, for predicting the yield of each DSB category as a function of proton dose and LET_t_

	Simp DSB	SimpBase DSB	Comp DSB	CompBase DSB
*a*	(–2.44 ± 0.36) × 10^−3^	(−6.77 ± 1.46) × 10^−4^	(1.29 ± 0.28) × 10^−3^	(3.47 ± 0.21) × 10^−3^
*b*	(3.98 ± 0.12) × 10^−1^	(2.09 ± 0.1) × 10^−1^	(3.16 ± 0.01) × 10^−1^	(1.41 ± 0.00) × 10^−1^
*c*	(1.64 ± 0.01) × 10^1^	(2.38 ± 0.03) × 10^0^	(4.86 ± 0.05) × 10^0^	(1.56 ± 0.04) × 10^0^

### Clinically relevant considerations

**Fig. 6:**
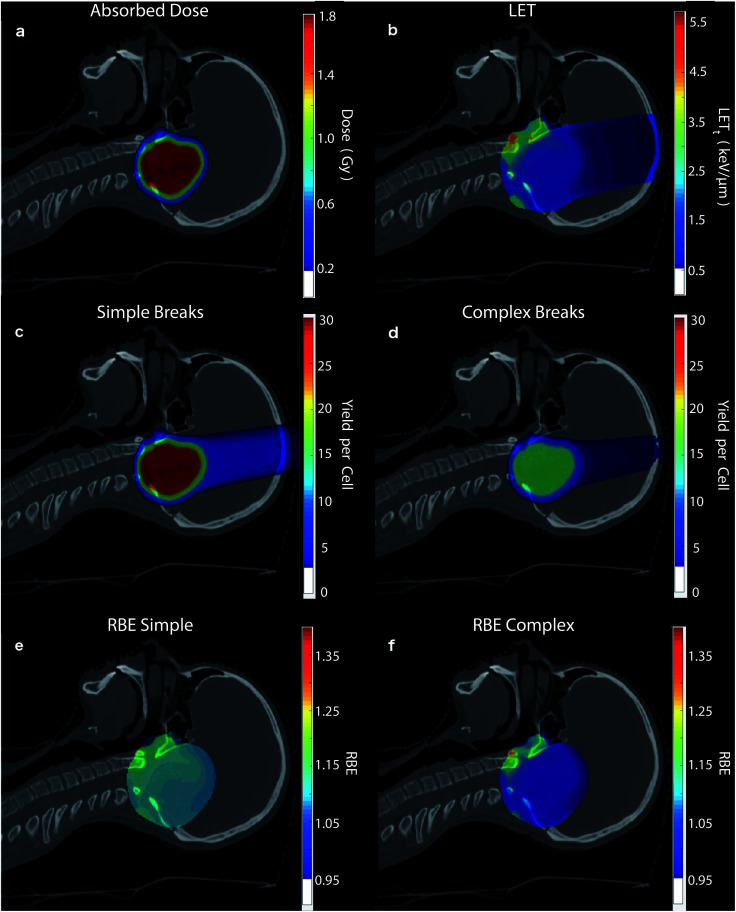
Using the correlations from this work the (a) proton physical dose (Gy) and (b) LET_t_ (keV μm^−1^) are used to predict (c) the expected yield of simple DSBs, involving only two damaged backbones, for cells in each voxel and (d) the expected yield of complex DSBs, involving either multiple damaged backbones or bases, for cells in each voxel. (e) RBE of simple damage, with the yield of simple breaks divided by the yields predicted for photons at the same physical dose and (f) the same but for complex breaks. Note, a threshold is applied to the colour bar, voxels below this given value aren't coloured (shown in the colour bar).

To highlight the clinical relevance of this modelling the correlations are applied to a typical treatment case of ependymoma, Fig. 6. A 3-field, single field optimised, Intensity Modulated Proton Therapy (IMPT) plan was created and optimised using Eclipse™ (version 13.7) treatment planning software (Varian, Palo Alto). In the case shown 1.8 Gy of physical dose was prescribed to the target. The optimised spot weights were exported and the plan was simulated in the Geant4 based toolkit GATE,^69^ using the “QGSP_BIC” physics list. The process of exporting the treatment plan from Eclipse™ and simulating in GATE was handled by our in-house software, AutoMC. Here, both dose (Fig. 6a) and LET_t_ (Fig. 6b) were scored in each 2 × 2 × 2 mm voxel. The correlations were applied to calculate the expected average yield of simple DSBs (Fig. 6c) and complex DSBs (Fig. 6d) for cells located within each voxel. Simple DSBs and complex DSBs are shown schematically in Fig. 2d and e–g respectively. By comparing to the types of DSB produced by the same dose of Co-60 an RBE of damage is predicted, shown in Fig. 6e and f. Fig. 6e shows the ratio of yields for DSBs that contain two backbones only (RBE_Simple_). Fig. 6f shows the ratio of yields for DSBs that contain more than two backbones and or base damage (RBE_Complex_). An RBE of 1, in Fig. 6e and f, corresponds to an equivalence in the yields of damage between protons and photons. An RBE less than 1 corresponds to a greater induction of damage for photon irradiation.

## Discussion

This work presents the results of proton and photon induced DNA damage simulations, and predictions for the types of DNA damage produced by clinically relevant LET_t_. The mechanisms of direct and indirect DNA damage are investigated through simulation of dry plasmid experiments and irradiation of the chromatin fibre model. When combined with a model of the cell nucleus, predictions of the absolute yields for the DSB types are made. With simulation across a range of proton LET_t_ a series of correlations are produced and applied to a proton dose plan to make clinically relevant predictions of DSB types within the patient.

The results of the plasmid simulation, Fig. 3, show that the quarter cylinder DNA model with direct strand breaks determined by an energy range probability most closely reproduces experimental data. However, there is considerable variability in the experimental data. For example, the data from Vyšín *et al.*^65^ and Souici *et al.*^66^ are from experiments with the same plasmid (pBR322) and under similar conditions. Although the studies do not investigate the same LET_t_ range, extrapolation of the results would suggest disagreement in measured strand break yields. Without the specificity and agreement in the experimental data it is difficult to conclude whether the mechanism of direct damage has truly been understood. Instead, the assumptions that have been made, 10 bp damage separation and damage determined by an energy range probability, can only be described as conditions that produce reasonable results when compared to experiment. For example, recent work from Villagrasa *et al.*^70^ showed that using an energy threshold as the mechanism for direct strand break reproduces the experimental yield of 53BP1 foci. The impact of such parameters used for predicting DNA damage for electrons was recently presented by Lampe *et al.*^71^ Since the mechanism may not have been identified, applying these conditions to a larger, more complex, system requires caution. However, this does not negate the results presented in this work, since similar damage yields are predicted when compared to the literature data. Instead it highlights the need for more data to give confidence in the hypothesised mechanisms of direct DNA damage.

Similar statements can be made about the applied mechanism of indirect damage. The simulations presented in this work can reproduce the estimated 65% strand breakage from indirect damage induced by Co-60, Fig. 5. However, this methodology does not fully describe indirect damage. Here, the mechanisms of chemical reaction and DNA damage are combined into a single probability. A more detailed approach may involve simulations based on molecular dynamics,^72^ where the interaction of free radicals with DNA can be explicitly modelled with no prior assumptions. However, with current computational power this approach would severely limit the scale of the simulation. It is here that the chosen approach shows merit, allowing simulation of chemical diffusion in a system with appropriate size. The chosen approach is similar to recent work by Meylan *et al.*,^61^ although, in their work the authors separately model the action of chemical reaction and DNA damage. In our work we neglect the spectrum of chemical attacks that do not lead to DNA strand breaks. For example, our simulations do not account for DNA adducts. This type of scoring would be necessary to fully quantify DNA repair efficacy, and to investigate effects such as the oxygen fixation hypothesis.^37^ This consideration of indirect damage is particularly important when considering hypoxia and other non-ambient biological conditions. Our model considers the cell to be made of water and as such the yields and species of free radicals are described by water radiolysis. This is a common assumption in modelling studies,^45,47,73,74^ often justified by the abundance of water in the cell. As far as we are aware no study has assessed the impact of this assumption by comparing to results from a more realistic medium.

Fig. 4 shows that across the proton LET_t_ range investigated, the majority of strand breaks are formed from indirect action. This suggests that by not including indirect damage in simulations it is likely that the total yield of complex damages will be underestimated. Since the diffusion of the OH radical is on the order of nanometres, for the time scales investigated, the most likely effect is to add damage to a directly induced cluster. This is shown explicitly in Fig. 5, where simulating direct effects only results in a higher probability of simple DSBs. Analytically going from these direct DSBs to the types of DSBs produced when indirect effects are included is difficult, since there is no constant scaling factor. This would imply that scoring direct DSBs does not give a clear indication of the complexity when indirect effects are included. However, direct damage alone does reproduce the relative yields of DSB types below around 20 keV μm^−1^, with simple DSBs most likely and multi-backbone multi-base DSBs the least likely.

Our predictions of DNA damage, and complexity of damage, are consistent with data from other models (see ESI 1†). Values for the RBE of DSB induction (RBE_DSB_) between protons and photons are compared to predictions from MCDS,^75^ PARTRAC,^53^ and Pater *et al.*^76^ Our model predicts similar trends of RBE_DSB_ with LET compared to the literature data, though at a slightly higher magnitude (ESI 1, Fig. S1†). The predicted proportion of complex DSBs shows agreement when compared to predictions from Nikjoo *et al.*^77^ and Lampe *et al.*^78^ (ESI 1, Fig. S2†). Taken together, this comparison gives confidence in the prediction of damage yields and complexity. Experimentally the detection of complex DNA damage is more difficult (see the review by Nikitaki *et al.*^79^), relying largely on the colocalization of fluorescent foci. This technique was used by Nikitaki *et al.*^80^ to show an increase in clustering of DSB and non-DSB damage with LET, between photons, alpha particles, and argon ions. Carter *et al.*^21^ used a modified comet assay to show an increase in complex damage with LET, which the authors propose leads to a decrease in cell survival. Chaudhary *et al.*^81^ irradiated cells at varying depths across a proton spread-out Bragg peak, and therefore at a range of LET. Using 53BP1 as a DSB marker Chaudhary *et al.* show a slight, yet non-significant, increase in foci per cell with LET. This increase in foci does not correspond to the expected increase predicted by our work. However, it is important to remember that the link between 53BP1 foci formation and DSB induction is not necessarily one-to-one, due to initial repair, protein affinity, and microscope resolution. As another measure of damage complexity, Vyšín *et al.*^65^ investigated the impact of nearby base damages in irradiated plasmids through use of BER enzymes. The authors saw no effect of LET in the range investigated. One interpretation of this is that the higher LET did not result in more base damages proximal to backbone damage. However, the authors comment that the electrophoresis method used is not sensitive enough to distinguish between damage types at this level.

The LET_t_ and dose dependencies shown in Fig. 5 are used to give total DSB yield as well as the relative types of DSB (eqn (2)). Due to investigation across a large range of proton LET_t_ values correlations can be determined. These fits give no information on the underlying mechanism, but instead allow for prediction of DSB types as a function of LET_t_. The detail of these predictions can be furthered to estimate the number of backbones and bases involved in the cluster (see ESI 2†). All the fits developed in this work are used to make clinically relevant predictions for a proton treatment plan, Fig. 6. Here, proton dose and LET_t_ are scored per voxel. These conventional dosimetric units are then converted into biologically relevant values, giving the yields of DSB type across the treatment field. The predominant DSB type is the simple form, involving only two backbones. The yield of complex DSBs remains relatively constant across the plateau region, with a change of around ±10%. With such a small increase in complexity it is difficult to foresee this as being the cause for increased biological effectiveness, unless the small increase in complexity results in a significant increase in cell kill. This would then imply that the complex DSBs are very toxic to the cell. We do not assign toxicities to the types of breaks predicted, since we don't include a model of DNA repair, though this work is ongoing.^82^ When comparing the predicted induction of complex breaks between protons and photons the RBE_Complex_ increases from 0.95, at the entrance, to 1.47 at the distal edge (ESI 3†). These trends are largely consistent with some of the predictions made by phenomenological models of RBE for cell kill.^9^ This increase in RBE of damage is not as apparent in the clinical case (Fig. 6), where regions of heightened RBE are due to LET hotspots rather than increasing LET with depth. In Fig. 6, the RBE is affected by the averaging of LET from 3 fields, which mitigates increased LET at the end of range. It is important to note that the complexity defined in this work refers to a measure of the clustering of DNA damages forming the DSB, which in turn is a measure of the density of energy depositions. However, the model is capable of determining other metrics of complexity, such as the size and structure of the single strand overhangs of the DSB ends (see ESI 4†). As mechanisms of repair pathways are uncovered the model can be used to report on biologically relevant complexities.

The change in DSB complexity is accompanied by another mechanism, which, when considered together, can likely explain the experimentally observed increase in cell kill with LET. Our previous work^18^ has shown that DSBs are induced more proximal with increasing LET_t_. The closer proximity leads to a higher yield of misrepaired DSBs, some of which will lead to chromosome aberrations and increased toxicity. The relation between increasing LET and increasing complexity cannot be uncoupled from the relation between increasing LET and closer proximity between breaks.

There are biologically relevant damages that are not accounted for in this work, aside from the described break complexity and proximity. For example, as previously mentioned chemical attacks that lead to adducts are neglected here. It is possible that these types of damage could slow the repair process, or create irreparable damage,^37^ which would increase the biological effectiveness.

## Conclusions

In this work simplified DNA geometries were assessed for their applicability in predicting DNA damage through track structure simulation. The mechanisms of direct and indirect damage were investigated through the simulated irradiation of plasmids and the chromatin fibre. A combination of DNA geometry, direct damage mechanisms, and indirect damage mechanisms were determined to reproduce yields of damage reported in the literature. Namely, a quarter cylinder model of DNA, an energy range probability for direct damage, and a probability for OH interaction with DNA leading to a strand break. With these mechanisms, predictions of proton and photon induced clustered and non-clustered DNA damage were made. These predictions show that there is a slight increase in damage complexity with increasing LET_t_. Specifics of the DSB can be predicted as a function of LET_t_ alone, where the cumulative distribution function that describes the number of backbones and bases included in a DSB cluster can be reproduced with a 3-parameter correlation (ESI 2†). This allows for fast prediction of detailed DSB complexity without the need for track structure simulation. By including a model of the nucleus, predictions are made for the absolute yields of DSB type as a function of dose and LET_t_. Here, correlations are drawn and applied to a clinically relevant case of ependymoma treatment; similarly, correlations are drawn for cells irradiated by photons. By comparing the yields of DSB type for protons and photons at the same physical dose an RBE for damage is calculated. This RBE increases with proton depth (ESI 3†), since it is a function of LET_t_. The values predicted for RBE of damage are similar in trend and value to those predicted for RBE of cell death by the phenomenological models. This lends weight to the idea that cell kill is related to the spatial clustering of energy depositions, characterised in this work by the break complexity through the number of damaged DNA volumes in a cluster. In radiotherapy dose is used as a surrogate for biological effect, though the relationship is less than perfect. Here, we show instead directly the effect that combining dose and LET_t_ has on the DNA, predicting types of damage that will later manifest as biological effect. The data produced by this work can be further used as an input to biological models through use of a standard format,^83^ which may help to elucidate dependencies between the damage pattern and outcomes.

## Author contributions

N. T. H. developed the DNA damage model, gathered the data, and analysed the data. A. H. A. developed the software to automate Monte Carlo simulation of treatment planning software generated plans. E. A. K. S. simulated the data shown in Fig. 6. M. J. M. and K. J. K. provided day to day supervision. R. I. M. and A. H. A. provided clinical physics guidance. N. G. B. provided clinical application. N. F. K. supervised the mathematical modelling. A. L. C. provided information on the biological relevance. M. S. generated the 1D SOBP in Fig. S3.† J. W. W. and S. P. I. provided information relevant to DNA repair. J. W. W. aided in the development of the code used in this work. N. T. H. drafted the manuscript based on discussions with the authors. All authors reviewed and approved the manuscript.

## Conflicts of interest

There are no conflicts to declare.

## Supplementary Material

RA-009-C8RA10168J-s001
