# Supplementary material for: Clinically relevant nanodosimetric simulation of DNA damage complexity from photons and protons
Source: RSC Adv. 2019 Feb 28;9(12):6845–58. doi: 10.1039/c8ra10168j (PMC9061037; doi:10.1039/c8ra10168j)
Supplement: RA-009-C8RA10168J-s001 [file RA-009-C8RA10168J-s001.pdf]

Electronic Supplementary Material (ESI) for RSC Advances.
This journal is © The Royal Society of Chemistry 2019

Clinically relevant nanodosimetric simulation of DNA damage complexity from photons and protons

Henthorn, N T[1, 2, *] ; Warmenhoven, J W[1, 2] ; Sotiropoulos, M[1] ; Aitkenhead, A H[1, 3] ; Smith, E A K[1, 2] ; Ingram, S P[1, 2] ; Kirkby, N F[1, 2] ; Chadwick, A L[1, 2] ; Burnet, N G[1, 2] ; Mackay, R I[1, 3] ; Kirkby, K J[1, 2] ; and Merchant, M J[1, 2]
[1]Division of Cancer Sciences, School of Medical Sciences, Faculty of Biology, Medicine and Health, The University of Manchester, UK
[2]The Christie NHS Foundation Trust, Manchester Academic Health Science Centre, Manchester, UK
[3]Christie Medical Physics and Engineering, The Christie NHS Foundation Trust, Manchester, UK
[*]Correspondence to: nicholas.henthorn@manchester.ac.uk

1. Complexity Compared to Literature. The model predictions are compared to literature data, firstly for the Relative Biological Effectiveness (RBE) between protons and photons of Double Strand Break (DSB) induction (Figure S1) and secondly for the proportion of complex DSBs (Figure S2).

Figure S1 shows our model prediction for the RBE of DSB induction (RBE_{Total}), the RBE of simple DSB induction (RBE_{Simp}), and the induction of complex DSBs (RBE_{Comp}) across the investigated proton LET range. Literature data for DSB induction is extracted from Pater *et al.* [1], PARTRAC [2], and MCDS [3]. Our predictions for RBE_{Total} are slightly higher than the other data presented. The literature data replotted in Figure S1 doesn't provide details of DSB complexity.

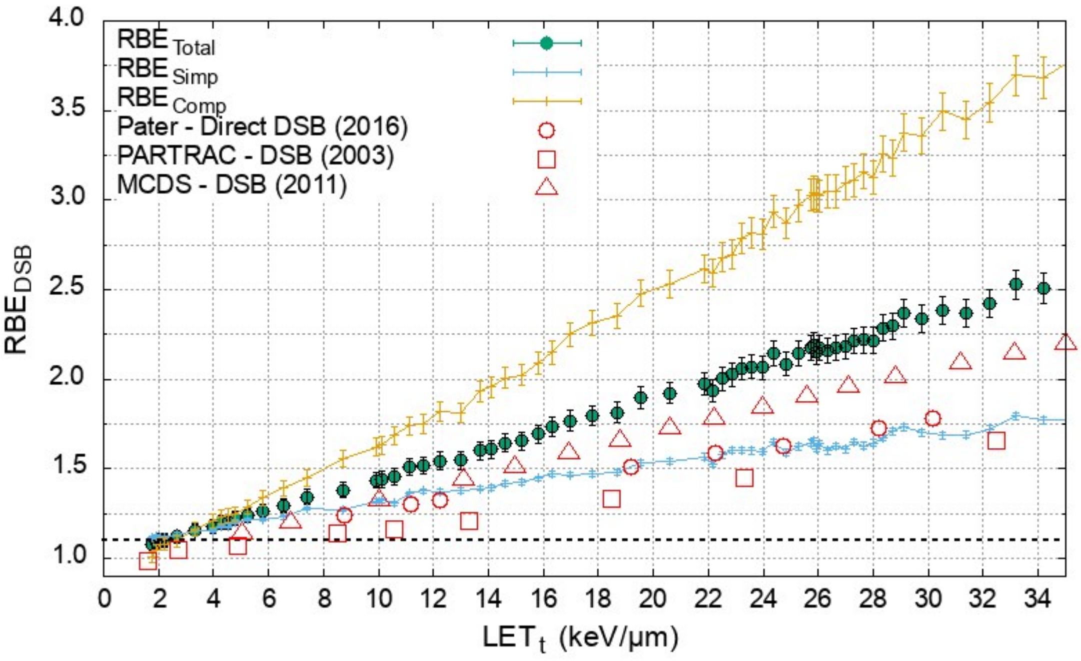

Figure S1. *The RBE for DSB induction (RBE_{Total}), simple DSB induction (RBE_{Simple}), and complex DSB induction ($RBE_{Complex}$) for our model. Compared to literature data for RBE_{Total} from Pater et al.* [1], PARTRAC [2], and MCDS [3]. Our predictions show similar trends in RBE with a slight increase in magnitude.

To compare DSB complexity, data is extracted from Nikjoo *et al.* [4] and Lampe *et al.* [5]. The DSB complexity in these works is categorised according to the scheme proposed by Nikjoo *et al.* [6]. Within this categorisation, complex breaks are referred to as "DSB+" and "DSB++". DSB+ are DSBs with one associated backbone damage, whilst DSB++ are DSBs with multiple associated backbone damages. Together DSB+ and DSB++ are equivalent to our category "complex DSB", shown in Figure 2f. It is important to note that the parameters of the Lampe model were chosen to reproduce the Nikjoo data. Our predictions for complex breaks agree with the extracted literature data.

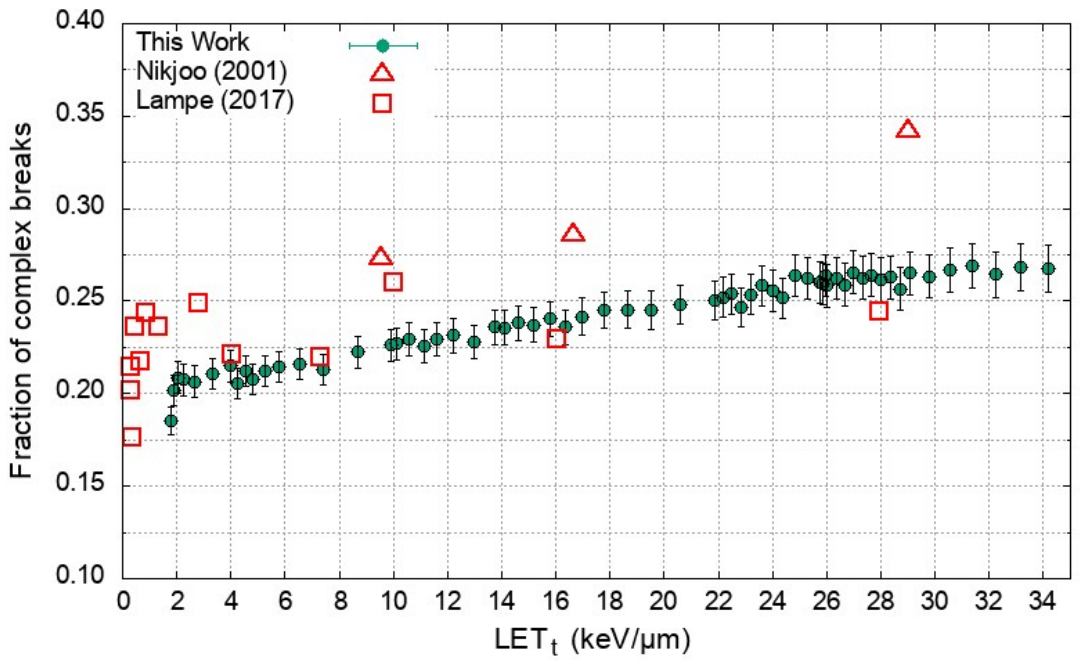

Figure S2. *The fraction of DSBs with one or more associated backbone damages for this work, the Nikjoo model [4], and the Lampe model [5].*

2. Damage Complexity Distribution. The data presented in this work describes the types of DNA damage produced across a proton LET$_t$ range. However, it may be of interest to determine the exact number of backbones and bases associated to a damage cluster. There is likely to be some variation in the biological response, even for breaks in the same category, e.g. a complex break with 5 damaged backbones may produce a different response than a complex break with 3 damaged backbones. The distribution of backbones and bases involved in a cluster is shown for a range of LET$_t$ (Figure S3), for the case of total damage mechanisms (both direct and indirect damage). These distributions are presented as cumulative distribution functions (CDF). Figure S3 presents some of these CDFs, with data selected to represent a range across the investigated LET$_t$. Figure S3 shows that the most likely cluster, for any proton LET$_t$, involves 2 backbones and 0 bases. The CDFs can be described by Equation S1, with 3 fitted parameters. This correlative equation is shown for the CDFs in Figure S3 with dashed lines. The parameters of Equation S1 are fit across the LET$_t$ range, shown in Figure S4.

$$CDF(\nu) = 1 - exp\left[\sqrt{\nu + a} - b(\nu + a)^n\right] \qquad (S1)$$

Where n and b are fitted parameters. ν is the cluster size, the number of bases or backbones in the cluster. a takes the value of 0 for the backbone distribution and 2 for the base distributions. Equation S1 has validity for cluster sizes $\nu \geq 2$ when describing the backbone distributions, and $\nu \geq 0$ when describing the base distributions.

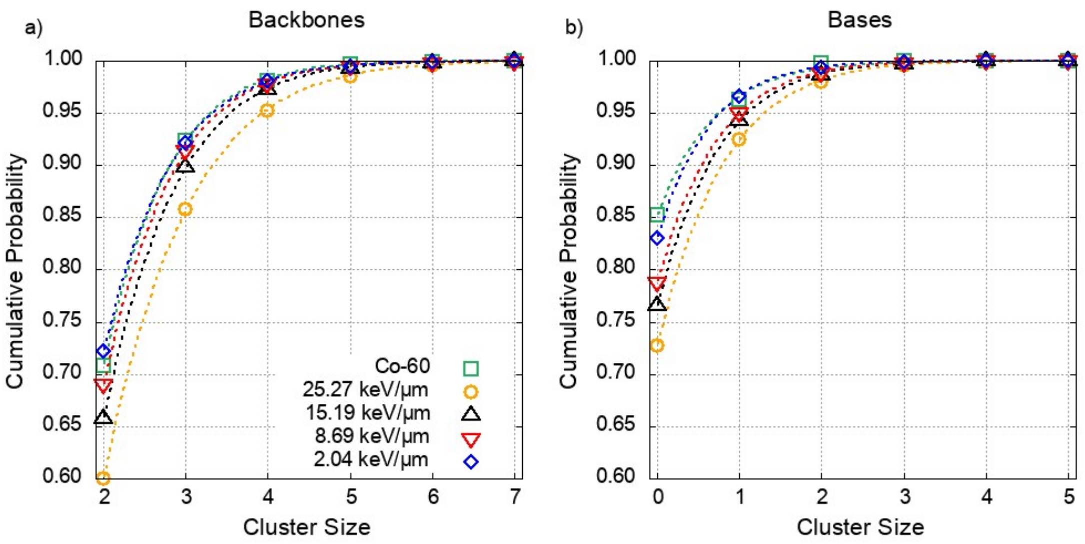

Figure S3. *The cumulative probability distribution of forming a cluster with a given number of* ***a)*** *backbones,* ***b)*** *bases. Dashed lines show Equation S1, with fitted parameters. Higher* LET_t *protons have a greater probability of inducing DSBs containing multiple damaged backbones and bases, leading to more complex breaks.*

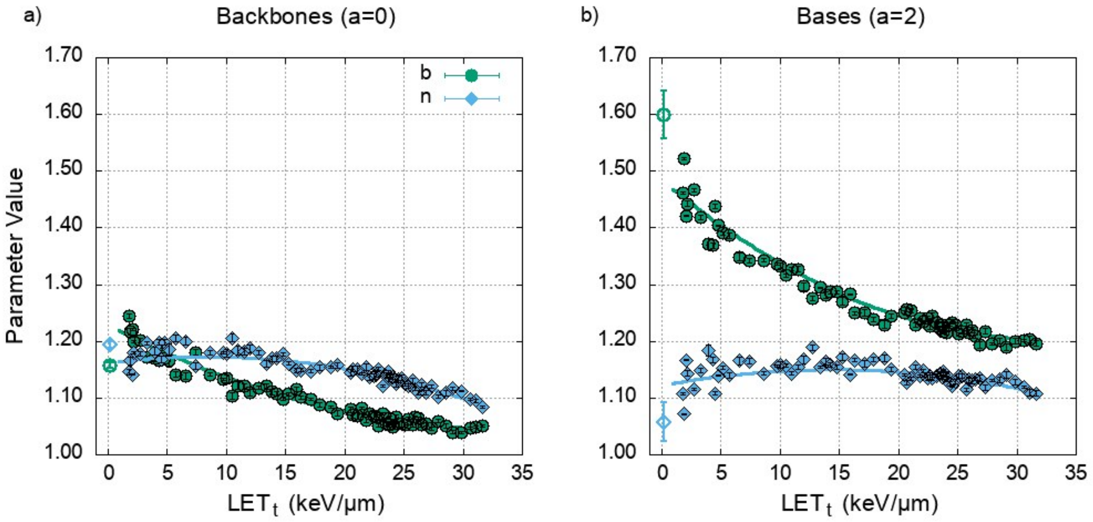

Figure S4. *The Cumulative Distribution Functions that describe the number of backbones and bases involved in a DSB can be determined by a 3-parameter fit (b, n, a). This figure shows the dependence of the fitted parameters on proton* LET_t *for* ***a)*** *backbones and* ***b)*** *bases. Parameters for the photon distribution fits are shown as open symbols at* $LET_t = 0.2$ *keV/µm. Error bars show the asymptotic standard error in the mean from the fit.*

Figure S3 shows how the specific break complexity varies with proton LET_t. Here, the number of backbones and bases involved in a DSB is shown as the cumulative probability. This CDF can be sampled to reproduce the DSBs predicted by the complex geometry and track structure simulations. The CDFs can be summarised by a 3-parameter correlation, Equation S1. Equation S1 can reproduce the CDF across the LET_t range investigated, with fitted parameters shown in Figure S4. The parameters (b or n) of the CDF fit can in turn be predicted as a function of LET_t, allowing for reproduction of the specific break size as a function of LET_t alone, Equation S2.

$$Parameter = f \times L^2 + g \times L + h \qquad (S2)$$

Where Parameter refers to either b or n in the cumulative distribution function, L is the track averaged LET and f, g, and h are parameters of the fit. The correlative parameters (f, g, h) that give the parameters for Equation S1 are presented in Table S1 for backbones and bases.

	b	n

	Backbones	Bases	Backbones	Bases
**f**	(1.57 ± 0.16) E-4	(2.67 ± 0.32) E-4	(-1.55 ± 0.20) E-4	(-1.34 ± 0.34) E-4
**g**	(-1.07 ± 0.05) E-2	(-1.73 ± 0.09) E-2	(2.57 ± 0.67) E-3	(3.86 ± 1.01) E+3
**h**	(1.23 ± 0.00) E+0	(1.48 ± 0.01) E+0	(1.16 ± 0.00) E+0	(1.12 ± 0.01) E+0

The fit becomes particularly noisy at low values of LET_t. The CDF fits presented in Figure S3 can be coupled to more simple damage simulations that do not explicitly model the DNA geometries, such as the cell model presented in this work. This allows for much faster simulation of DNA damage whilst retaining the same level of detail. For those interested only in break complexity, Equation 3 can be used to determine the spectrum of DSB type with no simulation, provided LET_t is known or assumed.

3. DSB complexity across a proton spread-out Bragg peak. The correlations derived in this work are used to predict damages across a proton SOBP, Figure S5, with 1 Gy across the dose plateau region. The SOBP is composed from 9 pristine Bragg peaks, with a maximum energy of 150 MeV, simulated with the Geant4 "QGSP_BIC" physics list. Here, it can be seen that across the entire proton dose depth profile the predominant DSB type is the simple form, involving two backbones only.

By comparing the types of DSB produced by Co-60 simulation an RBE for damage is predicted, shown in Figure S5c. The total yield of each damage type predicted for proton irradiation is divided by the yield predicted for the same physical dose of photons. RBE_{Total} is the ratio of the total yields of DSB, RBE_{Simple} is the ratio of yields for DSBs that contain two backbones only, and $RBE_{Complex}$ is the ratio of yields for DSBs that contain more than two backbones and or base damages.

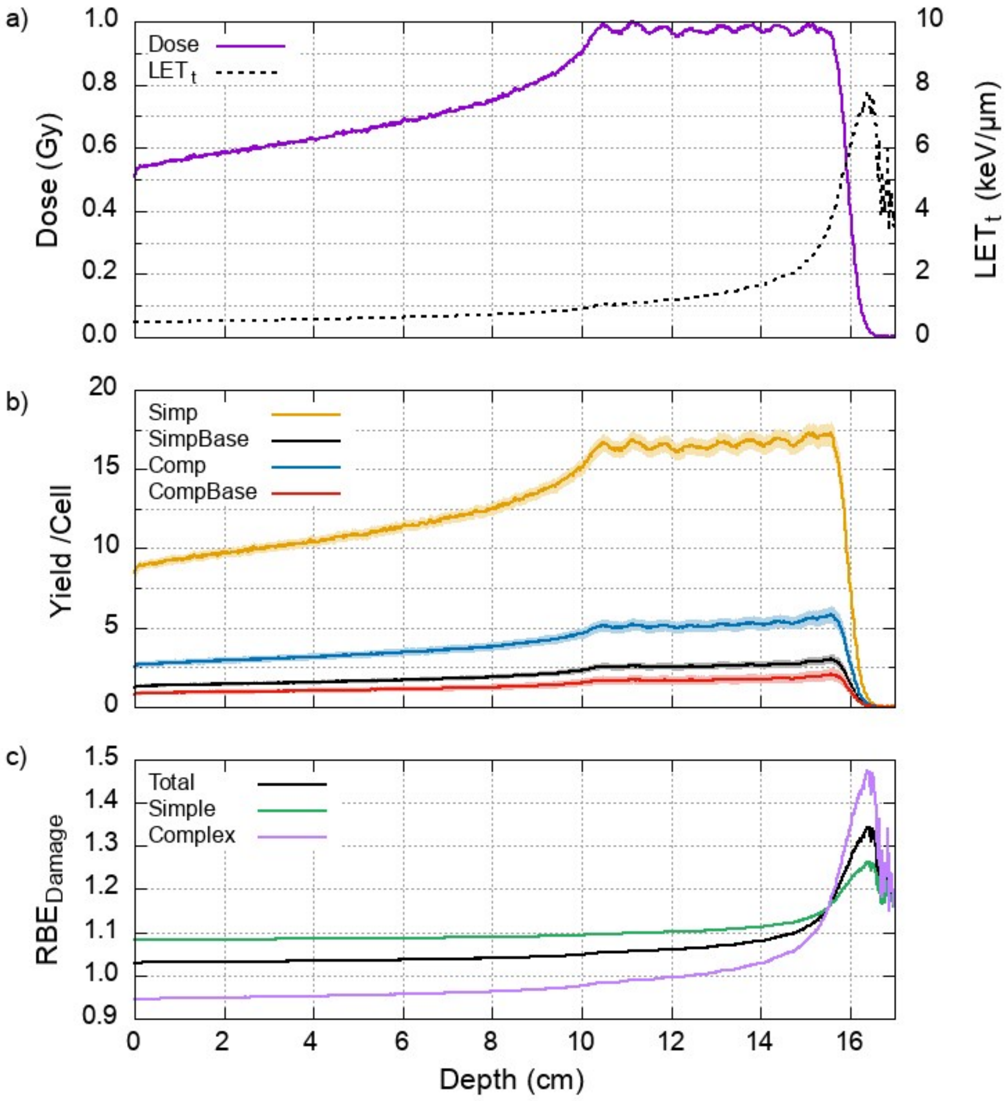

Figure S5. *Using the correlations from this work the* ***a)*** *proton physical dose and LET_t are used to predict the* ***b)*** *types of induced DSBs in a cell across a SOBP. Simple DSBs, involving only two backbones, are the dominant damage type*

across the entire depth profile. The shaded area shows uncertainties from ±1σ on the fitted parameters. ***c)*** *RBE of damage for the yield of total, simple, or complex breaks predicted from protons divided by the yields predicted for photons of the same physical dose.*

4. Double Strand Break Overhang. In this work the Double Strand Break (DSB) is formed by a cluster of damaged DNA backbones, Figure S6a. We assume that the double helix breaks in one position, and any nearby damages are associated to the corresponding DSB end, Figure S6b. Alternatively, the associated damages may lead to the loss of small DNA fragments, Figure S6c. These two breakage mechanisms can lead to significant differences in the size and structure of the DNA overhang. The structure of the overhang may be an important metric in determining repair pathway choice, since it can affect the loading of proteins.

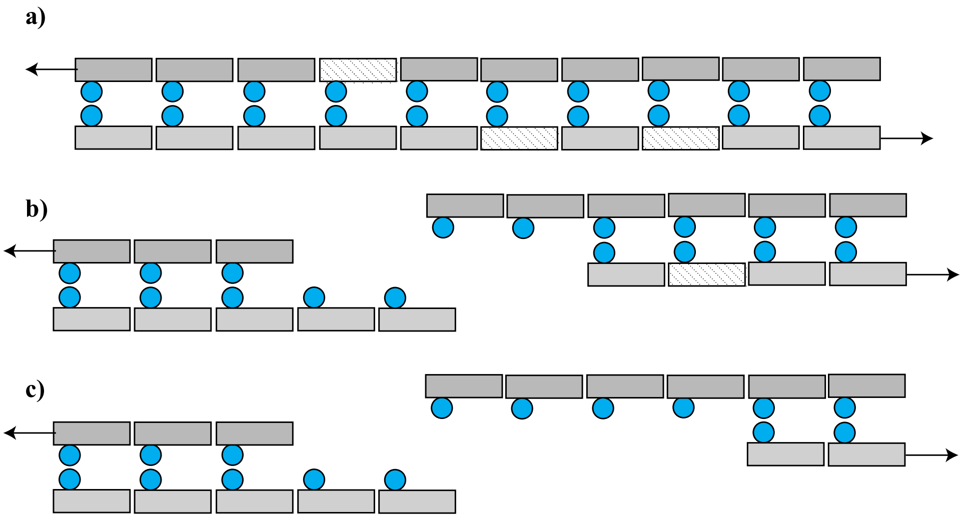

Figure S6. a) *An example of a DSB scored within this work.* ***b)*** *The structure of the DSB assuming nearby damages are associated to one of the break ends.* ***c)*** *The structure of the DSB assuming loss of DNA between nearby damages.*

The average overhang size, for the breakage mechanisms shown in Figure S6b and S6c, are shown across the proton LET range, Figure S7. Figure S7 shows a difference between the two breakage mechanisms. Assuming that the DNA only breaks in one location and that nearby breaks become associated to the DSB end, as in Figure S6c, results in a decrease in overhang size with LET. Here, the overhang size is determined by the closest pair of backbone damages on opposite strands. As the LET increases damages become denser, resulting in a smaller separation between opposing damages. Alternatively, assuming a loss of DNA between nearby damages, as shown in Figure S6c, results in an increasing overhang size with LET. Here, the overhang size is asymmetric for both DSB ends, with the overhang size determined by the farthest pair of damages. As the LET increases damages become denser, but also more frequent. The increased number of damages results in more damages clustering to form the DSB, reducing the separation between the closest pair of opposing damaged backbones but also increasing the separation between the farthest pair of damages.

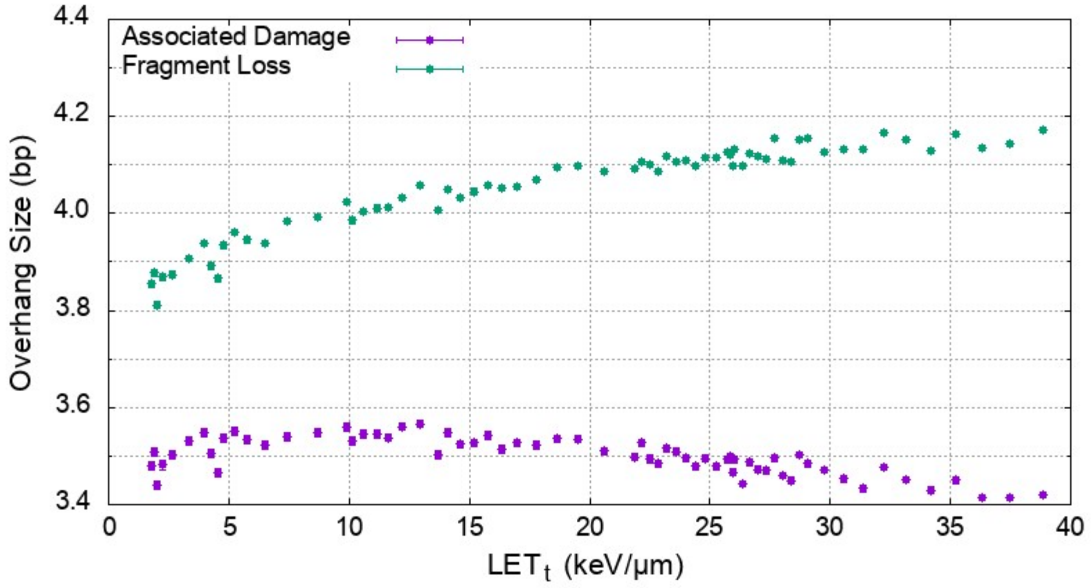

Figure S7. *The average size of the single stranded DNA overhang in base pair (bp). Calculated by assuming loss of DNA between associated backbone damages, as shown in Figure S6c (Fragment Loss), or assuming associated damages don't cause a loss of DNA, as shown in Figure S6b (Associated Damage). Error bars show the standard error in the mean between every DSB created over the 2500 repeats of 1 Gy.*

5. References

1. Pater, P. *et al.* Proton and light ion RBE for the induction of direct DNA double strand breaks. *Med. Phys.* **43,** 2131–2140 (2016).
2. Friedland, W., Jacob, P., Bernhardt, P., Paretzke, H. G. & Dingfelder, M. Simulation of DNA Damage after Proton Irradiation. *Radiat. Res.* **159,** 401–410 (2003).
3. Stewart, R. D. *et al.* Effects of radiation quality and oxygen on clustered DNA lesions and cell death. *Radiat. Res.* **176,** 587–602 (2011).
4. Nikjoo, H., Neill, P. O., Wilson, W. E. & Goodhead, D. T. Computational Approach for Determining the Spectrum of DNA Damage Induced by Ionizing Radiation. **583,** 577–583 (2001).
5. Lampe, N. *et al.* Mechanistic DNA Damage Simulations in Geant4-DNA Part 2: Electron and Proton Damage in a Realistic Cellular Geometry. *Phys. Medica* **in prep.,** 0–1 (2017).
6. Nikjoo, H., O'Neill, P., Goodhead, D. T. & Terrissol, M. Computational modelling of low-energy electron-induced DNA damage by early physical and chemical events. *Int. J. Radiat. Biol.* **71,** 467–483 (1997).
